# Treatment decisions in VRE bacteraemia: a survey of infectious diseases pharmacists

**DOI:** 10.1093/jacamr/dlad063

**Published:** 2023-05-22

**Authors:** Bryan P White, Katie E Barber, Daniel B Chastain

**Affiliations:** University of Oklahoma Medical Center, Department of Pharmacy, 700 NE 13th St, Oklahoma City, OK, USA; Department of Clinical Pharmacy, University of Mississippi College of Pharmacy, 2500 North State Street, USA; Department of Clinical and Administrative Pharmacy, University of Georgia College of Pharmacy, 1000 Jefferson Street, USA

## Abstract

**Background:**

VRE infections increased in 2020. High-dose daptomycin (≥10 mg/kg) has shown mortality benefit over other regimens, though daptomycin resistance is increasing. Limited data exist on the practice patterns of ID pharmacists for VRE bloodstream infections (VRE BSIs).

**Objectives:**

To describe practice patterns for VRE BSI in ID pharmacists.

**Methods:**

A 22-question REDCap survey was distributed to ID pharmacist members of the American College of Clinical Pharmacy (ACCP) Infectious Diseases Practice and Research Network (ID PRN) via e-mail listserv. The survey was distributed on 7 April 2022 and remained open for 4 weeks.

**Results:**

Sixty-eight pharmacists responded. All pharmacists completed additional training or certification in infectious diseases past their PharmD, and most (70.5%) had been practising for 10 years or less. Pharmacists at academic medical centres (80.0%) were more likely (*P* = 0.001) to have implemented the updated CLSI breakpoints than pharmacists at other types of institutions (55.2%). Daptomycin was the preferred drug for VRE BSI (92.6%), with 10 mg/kg (72.1%) being the preferred dose. Adjusted body weight was the most common weight (61.2%) used for obese patients. Fourteen days (76.1%) was the most common treatment duration for VRE BSI. Pharmacists defined persistent VRE BSI as 5 days (68.7%) after first blood culture.

**Conclusions:**

ID pharmacists overwhelmingly selected high-dose daptomycin for VRE BSI. There were variations in practice and response rate when selecting combination therapy, managing persistent bacteraemia, and treating patients with high daptomycin MICs or previous exposure to daptomycin.

## Background

*Enterococcus* spp. account for 11%–13% of all types of healthcare-associated infections (HAIs) among adult patients in the USA.^1^ In addition, *Enterococcus* spp. are the number one cause of central line-associated bloodstream infections (CLABSIs) in long-term acute care hospitals and haematology/oncology units.^1^ The emergence of antibacterial resistance among *Enterococcus* spp. poses significant challenges. More than 30% of HAIs caused by *Enterococcus* spp. are resistant to vancomycin,^2^ with a 14% increase in hospital-onset VRE infections occurring from 2019 to 2020.^3^

Though *Enterococcus faecalis* is isolated more often than *Enterococcus faecium*,^4^ more than 80% of *E. faecium* isolates display resistance to vancomycin^5^ through *vanA* or *vanB* genes, making treatment of serious infections caused by VRE increasingly difficult. As such, VRE bloodstream infections (VRE BSIs) are associated with mortality rates of up to 40%,^6^ which is increased with delayed active therapy.^7^ The VRE BSI treatment paradigm shifted in recent years, with literature initially showing mortality benefit with linezolid compared with low-dose daptomycin (<6 mg/kg)^8–10^. More recent papers have shown amortality benefit with high-dose daptomycin (10–12 mg/kg).^11–13^ Daptomycin resistance in VRE is increasing, with some centres reporting rates between 15% and 21% before the CLSI breakpoint change.^14^ Poor outcomes observed in patients with infections due to daptomycin resistant VRE emphasize the need for combination therapy approaches.^15^ Limited data show that adding a β-lactam, aminoglycoside, linezolid or tigecycline to high-dose daptomycin may be combinations for synergistic activity and may improve outcomes.^16^

Unlike *Staphylococcus aureus* bacteraemia (SAB) where multiple surveys of treatment preferences of infectious diseases (ID) pharmacists^17^ and ID physicians^18,19^ are available, similar data are not available for VRE BSI. SAB treatment surveys demonstrated variation among clinicians in different types of hospitals and areas of the country. It is unknown if pharmacists also exhibit significant variation in care of patients with VRE BSI. Our aim was to describe practice patterns of ID pharmacists for the management of VRE BSI.

## Methods

The methods were similar to our previous survey on SAB.^17^ All authors had previous experience with survey development. The authors developed the survey to focus on unanswered questions or topics surrounding VRE BSI based on a recent review of the literature for VRE BSI that demonstrated mortality advantages with daptomycin therapy, highlighted the uncertainties of managing daptomycin non-susceptible VRE BSI, and questioned the role of combination therapy. A 22-question REDCap survey^20,21^ (available as Supplementary data at *JAC-AMR* Online) was distributed to ID pharmacist members of the American College of Clinical Pharmacy (ACCP) Infectious Diseases Practice and Research Network (ID PRN) via e-mail listserv. The survey was designed to collect demographic characteristics of the respondents as well as information on VRE BSI treatment choice, dosing and the role of combination therapy. Survey questions were not mandatory and were multiple choice. When appropriate, they included a branched response logic, as well as a free-response option when ‘other’ was selected. The survey was distributed on 7 April 2022 and remained open for 4 weeks. A reminder e-mail was sent on 21 April 2022 and the survey closed on 4 May 2022. Descriptive statistics were calculated to characterize participants’ demographic and practice characteristics, in addition to diagnostic and treatment strategies. Data were analysed utilizing Pearson’s chi-squared or Fisher’s exact test, as appropriate. Modified Bonferroni was utilized, when appropriate. A two-sided *P* value of 0.05 was considered statistically significant. Statistical analyses were performed using SPSS (version 28.0, IBM, Armonk, NY, USA). This project was approved by the institutional review board at the University of Georgia as exempt (Study ID PROJECT00005385).

## Results

Sixty-eight pharmacists answered at least one question on the survey. The membership of the ACCP ID PRN, which includes clinicians and trainees, when the survey was sent was 1636 for a response rate of 4%. Most participants (89.7%) spent the majority of time in their job role on ID pharmacotherapy or stewardship activities. All pharmacists completed additional training past their PharmD or were board certified in ID or other specialties (see Table 1) and most (70.5%) had been practising for 10 years or less. The most common practice settings were academic medical centres (44.1%) or community teaching hospitals (41.2%) with even distribution across facilities with 500 or fewer beds and those with more than 500 beds. Southern USA (36.8%) and midwestern USA (30.9%) were the most common regions of practice. Almost all (97.1%) of pharmacists primarily cared for adult patients. Most pharmacists (66.2%) were at institutions utilizing the 2020 updated CLSI breakpoints for *E. faecium*, whereas 31% had not or were unsure (see Table 2). Less than one quarter (20.6%) of pharmacists felt sure that they saw an increase of VRE bacteraemia during the COVID-19 pandemic.

**Table 1.** Demographics

Question	Answers	*n* (%)
Training or advanced credentialling^a^ (*N* = 60)	ID PGY2	41 (68.3)
	ID fellowship	2 (3.3)
	BCIDP	40 (66.7)
	BCPS AQ-ID	7 (11.7)
	MAD-ID or SIDP stewardship certificate	16 (26.7)
	Other	5 (8.3)
Years in practice since terminal training (*N* = 68)	≤5	29 (42.6)
	6–10	19 (27.9)
	11–15	8 (11.8)
	16–20	6 (8.8)
	>20	6 (8.8)
Primary practice setting (*N* = 68)	Community hospital, non-teaching	7 (10.3)
	Community hospital, teaching	28 (41.2)
	Academic/university medical centre	30 (44.1)
	Veteran’s Affairs hospital	2 (2.9)
	Other	1 (1.5)
Size of institution, number of beds (*N* = 68)	<250	9 (13.2)
	251–500	25 (36.8)
	501–750	18 (26.5)
	>750	16 (23.5)
Region of practice (*N* = 68)	Southern USA	25 (36.8)
	Midwestern USA	21 (30.9)
	Western USA	8 (11.8)
	Northeastern USA	13 (19.1)
	International	1 (1.5)

a Respondents were able to select multiple options.

**Table 2.** VRE bacteraemia treatment

Question	Answers	*n* (%)
Institution implementation of 2020 CLSI breakpoints for *E. faecium* (*N* = 68)	Yes	45 (66.2)
	No	15 (22.1)
	Not sure	6 (8.8)
	EUCAST breakpoints	1 (1.5)
	Do not use CLSI breakpoints	1 (1.5)
Drug preference for VRE bacteraemia, assuming all susceptible (*N* = 68)	Daptomycin	63 (92.6)
	Linezolid	5 (7.4)
	Other	0 (0)
Daptomycin dose recommendation for VRE bacteraemia susceptibility pending, mg/kg (*N* = 68)	6	0 (0)
	8	12 (17.6)
	10	49 (72.1)
	12	5 (7.4)
	Other	2 (2.9)
Weight used for daptomycin in obese patients with VRE bacteraemia (*N* = 67)	Actual	21 (31.3)
	Adjusted	41 (61.2)
	Fixed dosing	3 (4.5)
	Other	2 (3.0)
Regimen for VRE bacteraemia, daptomycin MIC of 4 mg/L (susceptible dose-dependent) (*N* = 68)	Daptomycin	38 (55.9)
	Linezolid	17 (25.0)
	Combination	13 (19.1)
Daptomycin dose for VRE bacteraemia, daptomycin MIC of 4 mg/L (susceptible dose dependent), mg/kg (*N* = 38)	6	0 (0)
	8	4 (10.5)
	10	18 (47.4)
	12	16 (42.1)
	Other	0 (0.0)
Backbone drug for combination therapy for VRE bacteraemia, daptomycin MIC of 4 mg/L (susceptible dose-dependent) (*N* = 13)	Daptomycin	11 (84.6)
	Linezolid	1 (7.7)
	Other	1 (7.7)
Drug(s) in combination with backbone drug for combination therapy for VRE bacteraemia, daptomycin MIC of 4 mg/L (susceptible dose-dependent) (*N* = 13)	Ampicillin	11 (84.6)
	Ceftaroline	6 (46.2)
	Ceftriaxone	2 (15.4)
	Ertapenem	4 (30.8)
	Gentamicin	0 (0)
	Rifampicin	0 (0)
	Doxycycline	0 (0)
	Tigecycline	1 (7.7)
	Piperacillin/tazobactam	1 (7.7)
	Other	4 (30.8)
Intervening to change linezolid to daptomycin started over weekend for VRE bacteraemia (daptomycin MIC of 2 mg/L) (*N* = 67)	Yes	26 (38.8)
	No	41 (61.2)
Treatment duration VRE bacteraemia without endocarditis, days (*N* = 67)	7	6 (9.0)
	10	4 (6.0)
	14	51 (76.1)
	Other	6 (9.0)

Daptomycin was the preferred drug for VRE bacteraemia by almost all pharmacists (92.6%), with 10 mg/kg (72.1%) being the most commonly utilized dose. However, few pharmacists (38.8%) would intervene to change linezolid when started over the weekend for VRE bacteraemia. In obese patients, pharmacists used adjusted body weight (61.2%) most often when dosing daptomycin. Daptomycin was still preferred by 55.9% of respondents in patients with VRE bacteraemia and elevated daptomycin MICs (4 mg/L), followed by linezolid (25.0%) and combination therapy (19.1%). The most common daptomycin doses recommended for VRE bacteraemia with elevated MICs to daptomycin were 10 mg/kg (47.4%) and 12 mg/kg (42.1%). Fourteen days (76.1%) was the most common treatment duration for VRE bacteraemia.

Most pharmacists defined persistent VRE bacteraemia as 5 days of bacteraemia (68.7%). Development of on-therapy resistance (65.7%) and vasopressors (43.3%) were the most common patient-related factors to cause use of combination therapy in persistent VRE bacteraemia (see Table 3). Alternatively, most pharmacists would use combination therapy in persistent VRE bacteraemia and endocarditis in patients who are not surgical candidates, regardless of other factors (58.2%), as well as in patients with recurrent VRE bacteraemia previously treated with daptomycin (40.3%). Daptomycin (85%) was the most common backbone for combination therapy for VRE BSI, whereas ampicillin (85%) and ceftaroline (46%) were the most common additional agents used for combination therapy for VRE BSI (Table 2).

**Table 3.** Persistent VRE bacteraemia and endocarditis treatment

Question	Answers	*n* (%)
Defining persistent VRE bacteraemia (days of bacteraemia) (*N* = 67)	5	46 (68.7)
	7	15 (22.4)
	10	0 (0)
	Other	6 (9)
Patient factors to use combination therapy in persistent VRE bacteraemia (select all that apply) (*N* = 67)	Vasopressors	29 (43.3)
	Immunodeficiency	25 (37.3)
	Development of on-therapy resistance	44 (65.7)
	Combination therapy regardless of additional factors	12 (17.9)
	None of these factors	7 (10.4)
Patient factors to use combination therapy in persistent VRE bacteraemia with endocarditis who was not a surgical candidate (select all that apply) (*N* = 67)	Vasopressors	21 (31.3)
	Immunodeficiency	18 (26.9)
	Development of on-therapy resistance	28 (41.8)
	Combination therapy, regardless of additional factors	39 (58.2)
	None of these factors	5 (7.5)
Drug preference for recurrent VRE bacteraemia in a patient who previously got daptomycin pending susceptibilities (*N* = 67)	Daptomycin	23 (34.3)
	Linezolid	17 (25.4)
	Combination therapy	27 (40.3)

Pharmacists at academic medical centres (80.0%) were more likely (*P *= 0.001) to have implemented the updated CLSI breakpoints than pharmacists at other practice sites (55.2%). Overall, two-thirds (66.2%) of pharmacists were at hospitals that had implemented the updated breakpoints. There were no other statistically significant differences in VRE BSI treatment based on practice setting, years of practice, bed size or region of practice.

## Discussion

In the first survey describing ID pharmacists’ management of VRE BSI, daptomycin was the preferred agent in cases with pending susceptibilities or high daptomycin MICs, and as part of combination therapy. In patients with recurrent VRE BSI and previous daptomycin use, combination therapy was preferred over daptomycin monotherapy. The preference for daptomycin was not firm as 60% of pharmacists would not intervene to recommend a change from linezolid to daptomycin for VRE BSI. Our findings are important to show that daptomycin is the drug of choice for VRE BSI, with a mortality of 40%,^6^ increasing incidence of VRE BSI,^3^ and increasing incidence of daptomycin resistance in VRE BSI.^22,23^

Our survey also showed that the use of combination therapy for VRE BSI is not standardized. Development of on-therapy resistance was the most common reason for using combination therapy in persistent VRE BSI. Daptomycin resistance in VRE BSI is increasing and rates of up to 21% have been reported in cancer patients.^22,23^ Rapid diagnostic blood culture technologies should investigate including daptomycin resistance markers on their panels. Two-thirds of respondents were at institutions that have implemented the updated 2020 CLSI breakpoints for VRE, a rate that was significantly higher at academic medical centres. The new CLSI VRE breakpoints only report a susceptible dose-dependent category for MICs less than or equal to 4 mg/L of daptomycin for *E*. *faecium*. This breakpoint was established to encourage clinicians to use higher doses (>8 mg/kg) for patients with VRE BSI due to better drug exposure than the 4–6 mg/kg labelled doses, safety of higher doses, and improved clinical outcomes in patients with VRE BSI.^14,24^ In contrast, a 2019 survey on updated breakpoints by the College of American Pathology of approximately 700 labs showed that only 47% of labs had implemented an updated imipenem breakpoint for *Acinetobacter baumannii* from 2014.^25^ With our survey showing higher adoption of updated breakpoints than previous laboratory surveys,^25^ the impact of ID pharmacists’ collaboration on implementing up-to-date breakpoints should be investigated.

Daptomycin (≥6 mg/kg) has been shown to decrease mortality for VRE BSI in a multicentre retrospective and a multicentre prospective cohort study as compared with linezolid.^12,26^ Further studies have shown decreased mortality with higher daptomycin doses of up to 10–12 mg/kg as compared with 6 mg/kg.^11,13,26^ Consistent with that evidence, more than 90% of pharmacists in the survey preferred daptomycin over linezolid, and 80% chose daptomycin doses of 10–12 mg/kg. There is evidence for high-dose daptomycin having improved outcomes for VRE BSI as compared with lower doses, but limited data on high-dose daptomycin exist.^27^ The benefit of daptomycin over linezolid for VRE BSI has shown to be consistent in patients switched from linezolid to daptomycin,^28^ but most pharmacists in the survey would not switch therapy. Our results are similar to a Twitter survey (open to any individual) by David van Duin in March 2022 (*n* = 1001), which showed daptomycin 10 mg/kg as the standard of care in an immunosuppressed patient with VRE BSI by 59% of participants.^29^ In addition, 14 days was the most common choice for treatment duration of VRE BSI, which is consistent with findings from a Twitter survey (*n* = 565) in March 2022 where 14 days (56%) was the most common treatment duration for an immunosuppressed patient with a line-related VRE BSI and negative echocardiography.^30^ Fourteen days for VRE BSI is also in the range of 7–14 days recommended by the IDSA CLABSI guidelines as a treatment duration for enterococcal BSIs.^31^

Obesity in adults increased from 30.5% in 2000 to 41.9% in 2020 the USA.^32^ Although daptomycin is weight-based, its pharmacokinetics do not scale linearly with weight, and fixed-dose regimens for VRE BSI have been suggested.^33^ The evidence for daptomycin use in VRE BSI was established in patients with a median BMIs of 26 kg/m^2^ or weights of 50–60 kg, and are not representative of the increased obesity seen in clinical practice.^11,13,26^ Although retrospective data for combined Gram-positive infections have shown no changes in outcomes for using adjusted body weight for daptomycin dosing,^34^ specific studies are lacking for VRE BSI. Even with this limited evidence, most pharmacists preferred to use adjusted body weight for dosing daptomycin in obesity. Further research on the correct dosing strategy of daptomycin in obese patients with VRE BSI is needed.

The evidence for combination therapy for VRE BSI is limited to *in vitro* studies and case reports.^15^ Daptomycin combined with either ampicillin or ceftaroline were the most common regimens chosen by pharmacists in our survey. Our results on the treatment of VRE BSI, with daptomycin MICs of 4 mg/L with higher-dose daptomycin being the most common choice by pharmacists, contrasted with a Twitter poll by Sassine (*n* = 286)^35^ in May 2022 where linezolid (53%) was the most common choice of participants.

Our study is not without limitations. Our sample size (*n* = 68) was smaller than other previous surveys of the ACCP ID PRN,^17^ but similar in size to a survey of the ACCP ID PRN on outpatient parenteral antimicrobial therapy (OPAT) (*n* = 87).^36^ With the majority of respondents in our survey having 10 years or less of practice, it may have led to bias. The only demographic factor in our survey associated with practices was hospital type and implementation of the new CLSI breakpoints; our smaller sample size may have prevented us from finding differences. Our survey did not evaluate the issues with agreement for testing daptomycin MICs for *E. faecium*.^37^ The study by Chuang *et al*.^13^ showing decreased mortality in patients with VRE bacteraemia with daptomycin MICs of 4 mg/L who received at least 12 mg/kg daptomycin was published after our survey was conducted. Our results showing that pharmacists prefer daptomycin at 10 mg/kg for VRE BSI with daptomycin MICs of 4 mg/L may not reflect current practice.

In conclusion, our data demonstrated that ID pharmacists prefer high-dose daptomycin for the treatment of VRE bacteraemia. There is variation in practice with regard to combination therapy, persistent bacteraemia and treatment of patients with high daptomycin MICs or previous exposure to daptomycin.

## Supplementary Material

dlad063_Supplementary_Data

## Supplementary data

The REDCap Survey is available as Supplementary data at *JAC-AMR* Online.
