# Supplementary material for: Treatment decisions in VRE bacteraemia: a survey of infectious diseases pharmacists
Source: JAC Antimicrob Resist. 2023 May 22;5(3):dlad063. doi: 10.1093/jacamr/dlad063 (PMC10202426; doi:10.1093/jacamr/dlad063)
Supplement: dlad063_Supplementary_Data [file dlad063_supplementary_data.docx]

**VRE bacteremia survey**

*Page 1*

The purpose of this study is to describe practice patterns of clinical pharmacists for the management of vancomycin resistant enterococcal (VRE) bacteremia. Our results could prove beneficial to design future educational or research efforts to improve care of patients with VRE bacteremia by infectious diseases pharmacists.

Your participation in this study is completely voluntary and you can refuse to participate or withdraw at any time. Your decision to participate or not will have no bearing on your grades, class standing or employment status. Completing the surveys implies consent to participate in the study. The University of Georgia Institutional Review Board determined that this study is not research involving human subjects as defined by DHHS and FDA regulations

Thank you in advance for your time and participation.

| Does ID pharmacotherapy and/or ASP activities comprise | Yes |  |
| --- | --- | --- |
| at least 50% of your current job role? | No |  |
|  |  |  |
| What training or advanced credentialing did you | ID PGY2 |  |
| complete (select all that apply)? | ID fellowship |  |
|  | BCIDP |  |
|  | BCPS AQ-ID |  |
|  | MAD-ID or SIDP stewardship certificate training |  |
|  | Other |  |
|  |  |  |
| What other training or advanced credentialing did you | __________________________________ |  |
| complete? |  |  |
|  |  |  |
| Since completion of terminal training, how many years | < or = 5 |  |
| have you been practicing? | 6-10 |  |
|  | 11-15 |  |
|  | 16-20 |  |
|  | > 20 |  |
|  |  |  |
| What is your primary practice setting? | Community hospital, non-teaching |  |
|  | Community hospital, teaching |  |
|  | Academic/University Medical Center |  |
|  | Infectious Diseases Clinic |  |
|  | Veteran's Affairs Hospitals |  |
|  | Other |  |
|  |  |  |
| What other type of practice setting? | __________________________________ |  |
|  |  |  |
|  |  |  |
| What is the size of the institution in which you | < 250 beds |  |
| practice? | 251-500 beds |  |
|  | 501-750 beds |  |
|  | > 750 beds |  |
|  |  |  |
| In which region of the world do you practice? | Southern U.S. |  |
|  | Midwestern U.S. |  |
|  | Western U.S. |  |
|  | Northeastern U.S. |  |
|  | International |  |
|  |  |  |
| Do you primarily take care of patients who are 18 | Yes |  |
| years and older? | No |  |


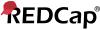

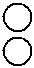

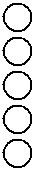

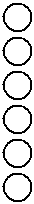

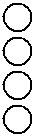

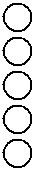

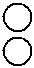


12/07/2022 8:31pm [projectredcap.org](https://projectredcap.org)

*Page 2*

| Has your institution implemented the CLSI breakpoints | Yes |  |
| --- | --- | --- |
| released in 2019 for Enterococcus faecium blood | No |  |
| isolates where daptomycin MICs ≤ 4 mg/L are listed | Not sure |  |
| as susceptible dose dependent? | We use EUCAST breakpoints |  |
|  | We don't use CLSI breakpoints |  |
|  |  |  |
| What drug do you prefer for Vancomycin resistant | Daptomycin |  |
| Enterococcus faecium bacteremia assuming all options | Linezolid |  |
| are susceptible? | Other |  |
|  |  |  |
| What other drug do you prefer for Vancomycin resistant | __________________________________ |  |
| Enterococcus faecium bacteremia assuming all options |  |  |
| are susceptible? |  |  |
|  |  |  |
| What dose of daptomycin do you normally recommend for | 6 mg/kg |  |
| Vancomycin resistant Enterococcus faecium bacteremia | 8 mg/kg |  |
| pending susceptibility data (i.e. being called with | 10 mg/kg |  |
| rapid diagnostic data)? | 12 mg/kg |  |
|  | Other |  |
|  |  |  |
| Enter other dose of daptomycin in mg/kg you normally | __________________________________ |  |
| recommend for Vancomycin resistant Enterococcus |  |  |
| faecium bacteremia pending susceptibility data (i.e. | (Please enter the mg/kg dose) |  |
| being called with rapid diagnostic data) |  |  |
|  |  |  |
| What weight do you use when dosing daptomycin for | Actual |  |
| Vancomycin resistant Enterococcus faecium bacteremia | Adjusted |  |
| in obesity (BMI ≥30 kg/m2)? | Fixed dosing |  |
|  | Other |  |
|  |  |  |
| What regimen do you use for Vancomycin resistant | Daptomyin |  |
| Enterococcus faecium bacteremia with a daptomycin MIC | Linezolid |  |
| of 4 mg/L (susceptible dose dependent)? | Combination therapy |  |
|  |  |  |
| What dose of daptomycin do you normally recommend for | 6 mg/kg |  |
| Vancomycin resistant Enterococcus faecium bacteremia | 8 mg/kg |  |
| with a daptomycin MIC of 4 mg/L (susceptible dose | 10 mg/kg |  |
| dependent)? | 12 mg/kg |  |
|  | Other |  |
|  |  |  |
| Enter the other dose of daptomycin in mg/kg you | __________________________________ |  |
| normally recommend for Vancomycin resistant |  |  |
| Enterococcus faecium bacteremia with a daptomycin MIC | (Please enter the mg/kg dose) |  |
| of 4 mg/L (susceptible dose dependent) |  |  |
|  |  |  |
| What drug do you normally use as a backbone for | Daptomycin |  |
| combination therapy for Vancomycin resistant | Linezolid |  |
| Enterococcus faecium bacteremia? | Other |  |
|  |  |  |
| What other drug do you normally use as a backbone for | __________________________________ |  |
| combination therapy for Vancomycin resistant |  |  |
| Enterococcus faecium bacteremia? |  |  |


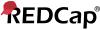

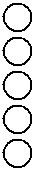

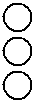

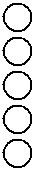

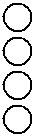

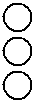

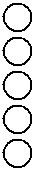

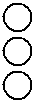


12/07/2022 8:31pm [projectredcap.org](https://projectredcap.org)

*Page 3*

| What drug(s) do you normally use in combination with | Ampicillin |  |
| --- | --- | --- |
| the previously identified backbone therapy? | Ceftaroline |  |
|  | Ceftriaxone |  |
|  | Ertapenem |  |
|  | Gentamicin |  |
|  | Rifampin |  |
|  | Doxycycline |  |
|  | Tigecycline |  |
|  | Piperacillin/tazobactam |  |
|  | Other |  |
|  |  |  |
| How long do you normally treat Vancomycin resistant | 7 days |  |
| Enterococcus faecium bacteremia without endocarditis? | 10 days |  |
|  | 14 days |  |
|  | Other |  |
|  |  |  |
| How long do you normally treat Vancomycin resistant | __________________________________ |  |
| Enterococcus faecium bacteremia without endocarditis? |  |  |
|  | (Please enter the number of days) |  |
|  |  |  |
| You come in on Monday and see that a patient is on | Yes |  |
| linezolid for Vancomycin resistant Enterococcus | No |  |
| faecium bacteremia with a daptomycin MIC of 2 mg/L |  |  |
| (susceptible dose dependent). Do you intervene to |  |  |
| change this patient's therapy to daptomycin? |  |  |
|  |  |  |
| How do you define persistent Vancomycin resistant | 5 days of bacteremia |  |
| Enterococcus faecium bacteremia? | 7 days of bacteremia |  |
|  | 10 days of bacteremia |  |
|  | Other |  |


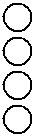

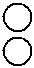

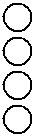


Which of the following factors would prompt you to select combination therapy in a patient with persistent VRE bacteremia (select all that apply)?


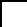

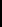
 Vasopressors


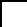

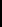
 Immunodeficiency


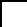

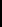
 Development of on therapy resistance


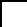

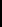
 Combination therapy regardless of additional factors


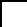

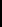
 None of these factors would prompt me to use combination therapy (only select this option if none)

Which of the following factors would prompt you to select combination therapy in a patient with persistent VRE bacteremia and infective endocarditis who is not a surgical candidate (select all that apply)?


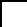

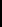
 Vasopressors


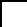

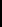
 Immunodeficiency


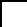

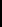
 Development of on therapy resistance


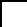

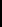
 Combination therapy regardless of additional factors


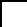

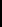
 None of these factors would prompt me to use combination therapy (only select this option if none)

| What drug do you prefer for recurrent Vancomycin | Daptomycin |
| --- | --- |
| resistant Enterococcus faecium bacteremia in a patient | Linezolid |
| who previously received daptomycin pending | Combination therapy |
| susceptibilities? |  |
|  |  |
| Have you observed an increase in the incidence of | Yes |
| vancomycin resistant Enterococcus faecium bacteremia | No |
| during the COVID-19 pandemic? | Unsure |


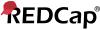

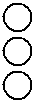

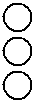


12/07/2022 8:31pm [projectredcap.org](https://projectredcap.org)
